# Bioinformatics Analysis Reveals Most Prominent Gene Candidates to Distinguish Colorectal Adenoma from Adenocarcinoma

**DOI:** 10.1155/2018/9416515

**Published:** 2018-08-06

**Authors:** Nina Hauptman, Emanuela Boštjančič, Margareta Žlajpah, Branislava Ranković, Nina Zidar

**Affiliations:** Institute of Pathology, Faculty of Medicine, University of Ljubljana, Ljubljana, Slovenia

## Abstract

Colorectal cancer (CRC) is one of the leading causes of death by cancer worldwide. Bowel cancer screening programs enable us to detect early lesions and improve the prognosis of patients with CRC. However, they also generate a significant number of problematic polyps, e.g., adenomas with epithelial misplacement (pseudoinvasion) which can mimic early adenocarcinoma. Therefore, biomarkers that would enable us to distinguish between adenoma with epithelial misplacement (pseudoinvasion) and adenoma with early adenocarcinomas (true invasion) are needed. We hypothesized that the former are genetically similar to adenoma and the latter to adenocarcinoma and we used bioinformatics approach to search for candidate genes that might be potentially used to distinguish between the two lesions. We used publicly available data from Gene Expression Omnibus database and we analyzed gene expression profiles of 252 samples of normal mucosa, colorectal adenoma, and carcinoma. In total, we analyzed 122 colorectal adenomas, 59 colorectal carcinomas, and 62 normal mucosa samples. We have identified 16 genes with differential expression in carcinoma compared to adenoma:* COL12A1*,* COL1A2*,* COL3A1, DCN, PLAU, SPARC, SPON2, SPP1*,* SULF1*,* FADS1, G0S2, EPHA4, KIAA1324*,* L1TD1, PCKS1*, and* C11orf96*. In conclusion, our* in silico* analysis revealed 16 candidate genes with different expression patterns in adenoma compared to carcinoma, which might be used to discriminate between these two lesions.

## 1. Introduction

Colorectal cancer (CRC) is developed by multistep process from normal epithelium to adenoma and adenocarcinoma, which can eventually metastasize to different organs [1]. The model of development of CRC was introduced in 1990, where* APC*,* KRAS*,* TP53*, and* DCC* were proposed as genes promoting the progression of CRC [2]. Since, many studies have investigated underlying molecular mechanisms of CRC. It is accepted that CRC arises from accumulation of genetic and epigenetic events that alter signaling in pathways, such as Wnt, PIK3CA, and TGF-*β*. Three major accepted pathways in the pathogenesis of CRC are chromosome instability pathway, microsatellite instability pathway, and CpG island methylator phenotype. There are many CRCs that lack the changes described in above pathways, suggesting that other mechanisms are involved in the development of CRC [1].

CRC is one of the leading causes of death by cancer worldwide. In Europe, CRC is the second and the third cause of death by cancer in men and women, respectively [3]. Five-year survival for patients with early CRC is 90%, while for patients with advanced CRC, survival drops to only 8-12% [4]. The prognosis can improve significantly with the introduction of population screening. Bowel cancer screening programs enable us to detect early lesions, including adenomas and adenomas with early adenocarcinoma (malignant polyps). However, they also generate a significant number of problematic polyps which contain dysplastic glands in the submucosa. This phenomenon has been referred to as epithelial misplacement (pseudoinvasion). It can be the result of a torsion or intraluminal trauma of large pedunculated polyps of the distal colon, or it may be a consequence of a previous biopsy. Adenomas with epithelial misplacement (pseudoinvasion) can be difficult to distinguish from adenomas with early adenocarcinoma [5–7]. The correct diagnosis is crucial for the choice of optimal treatment. For adenoma and adenoma with epithelial misplacement, endoscopic removal is sufficient, whereas malignant adenomas (early carcinomas) may require surgical treatment, since they are capable of metastasizing [7].

Despite well-defined morphologic features of epithelial misplacement and early invasion, there are a significant number of lesions with ambiguous features leading to divergent diagnostic opinions among pathologists [7]. Biomarkers that would enable to distinguish between adenoma with epithelial misplacement (pseudoinvasion) and adenoma with early adenocarcinoma (true invasion) are needed. We hypothesized that the former is genetically similar to adenoma and the latter to adenocarcinoma and we used bioinformatics approach to search for candidate genes that might be potentially used to distinguish between the two lesions.

Gene expression in CRC was widely studied by microarray technique, usually comparing carcinomas to normal mucosa tissue, studying microsatellite instable CRC, or establishing CRC subtypes based on gene expression patterns [8–10]. Some of the studies have focused on the gene expression difference between colorectal adenomas and carcinomas [11–15]. The downside of these studies is limitation in number of samples. Our goal was to minimize any variabilities arising from different microarrays and procedures, to identify the genes and subsequently pathways associated with adenoma progression to carcinoma. Due to the aim of the study, we have chosen five different sets of data, containing normal, adenoma, and carcinoma samples, where two of them were not published yet.

## 2. Materials and Methods

### 2.1. Microarray Data

Several projects (GSE10714, GSE37364, GSE41657, GSE50114, and GSE50115) with gene expression profiles of colon normal, adenoma, and carcinoma samples were downloaded from the public functional genomics data repository-Gene Expression Omnibus database (GEO, http://www.ncbi.nlm.nih.gov/geo) of the National Center for Biotechnology Information (NCBI). In total, 7 CRC, 5 adenomas, and 3 normal mucosa specimens were included in GSE10714, while 27 CRC, 29 adenomas, and 38 normal mucosa specimens were included in GSE37364 (both on platform GPL570 Affymetrix Human Genome U133 Plus 2.0 array). GSE41657 was composed of 25 CRC, 51 adenomas, 12 normal mucosa samples, and GSE50114 combined with GSE50115 contained 9 CRC, 37 adenoma, and 9 normal mucosa samples (all three on platform GPL6480 Agilent Whole Human Genome Microarray 4x44K G4112F). In total, 252 samples of colonic biopsies, including 62 normal, 122 adenomas, and 59 CRC samples, were included in this study.

### 2.2. Data Processing

For all projects, the original data files were downloaded and further normalized in R language (https://www.r-project.org/). For projects on Affymetrix arrays (GSE10714, GSE37364) package affy was used to convert CEL files into expression data using robust multichip average function, which performs background correction and normalization in one step [16]. For projects on Agilent arrays (GSE41657, GSE50114, and GSE50115) package limma was used to perform background correction and normalization between arrays [17]. After data normalization gene filter was used to remove probes that had intensity less than 100 in more than 20% of samples in each project.

Differentially expressed genes (DEG) were identified on probe level using limma package in R for each individual project [18]. We constructed three contrast matrices (adenoma compared to normal, carcinoma compared to adenoma, and carcinoma compared to normal) for each GEO project. The cut-off conditions were set to adjusted p value < 0.05 and absolute value of log fold change (log FC) > 1.5. Every comparison (adenoma compared to normal, carcinoma compared to adenoma, and carcinoma compared to normal) was overlapped among the projects to obtain the DEGs common to all projects.

### 2.3. Functional Analysis and Protein-Protein Interactions Network

For functional analysis and construction of protein-protein interactions (PPI) network, the Search Tool for the Retrieval of Interacting Genes (STRING) database was employed (https://string-db.org/). PPI network analysis is one of the important tools for interpretation of molecular mechanisms in the process of carcinogenesis. STRING offers integrative tools for uncovering the biological meaning behind large sets of genes, providing besides constructing PPI networks and also functional and pathway enrichment analysis. Gene ontology (GO) analysis including biological process, molecular function, and cellular component and Kyoto Encyclopedia of Genes and Genomes (KEGG) pathway enrichment analysis were conducted for selected DEGs with STRING. The statistical significance threshold was set to p < 0.05.

In this study, we constructed PPI networks of DEGs for carcinoma compared to normal, adenoma compared to normal, and carcinoma compared to adenoma. The PPI network was constructed under the cut-off of interaction score of 0.4. Visualization of all three networks together was done in Cytoscape version 3.5.1 (http://www.cytoscape.org/).

## 3. Results

**Figure 1:**
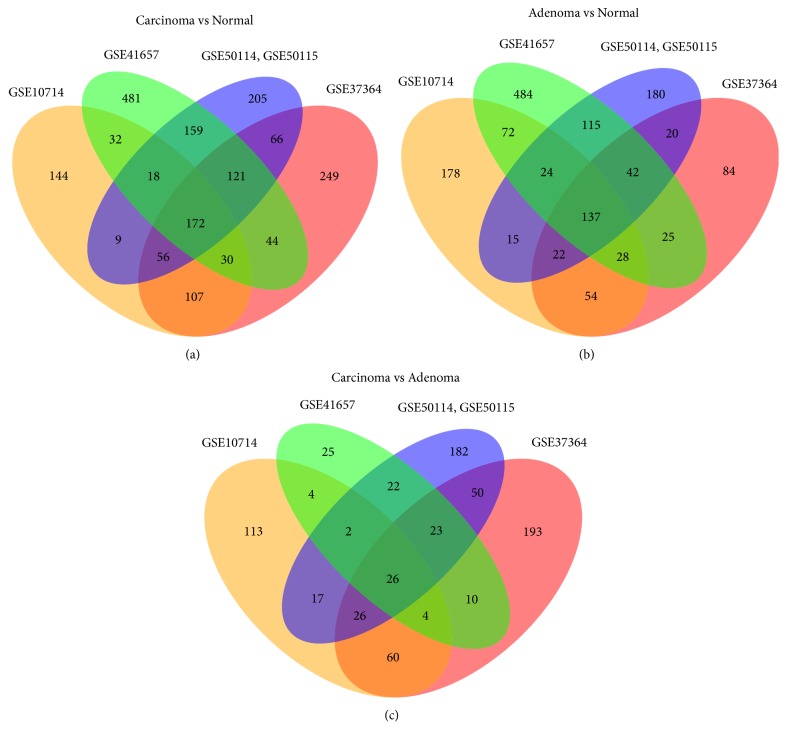
Identification of differentially expressed genes in gene expression datasets (GSE10714, GSE37364, GSE41657, GSE50114, and GSE50115) and their overlaps.

**Figure 2:**
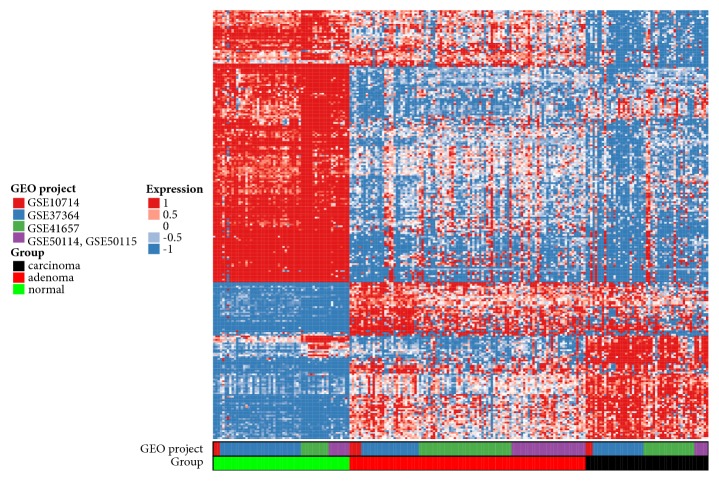
Heatmap of union of genes differentially expressed in each dataset (GSE10714, GSE37364, GSE41657, GSE50114, and GSE50115).

Data from each microarray was separately analyzed to obtain DEGs for each comparison, carcinoma compared to normal, adenoma compared to normal, and carcinoma compared to adenoma. We identified 172 genes overlapping in all projects for carcinoma compared to normal (568 in GSE10714, 845 in GSE37364, 1057 in GSE41657, and 806 in GSE50114 combind with GSE50115), 137 genes overlapping in all projects for adenoma compared to normal (530 in GSE10714, 412 in GSE37364, 927 in GSE41657, and 555 in GSE50114 combind with GSE50115), and 26 genes overlapping in all projects for carcinoma compared to adenoma (252 in GSE10714, 392 in GSE37364, 116 in GSE41657, and 348 in GSE50114 combind with GSE50115) (Figure 1). We also constructed heatmap with union of all genes differentially expressed in every individual project, to confirm that samples belong to three distinct groups, namely, carcinoma, adenoma, and normal mucosa samples (Figure 2).

In order to investigate our selected DEGs, we overlapped the genes in each comparison, to obtain the unique set of genes characteristic for each comparison (Supplementary Figure 1). As expected, the most DEGs were found in carcinoma compared to normal mucosa group (172), somewhat less in adenoma compared to normal group (137), and just 26 DEGs in carcinoma compared to adenoma group (Supplementary Table 1). Interestingly, there were no DEG common to all three comparisons.

### 3.1. Protein-Protein Interaction Networks

**Figure 3:**
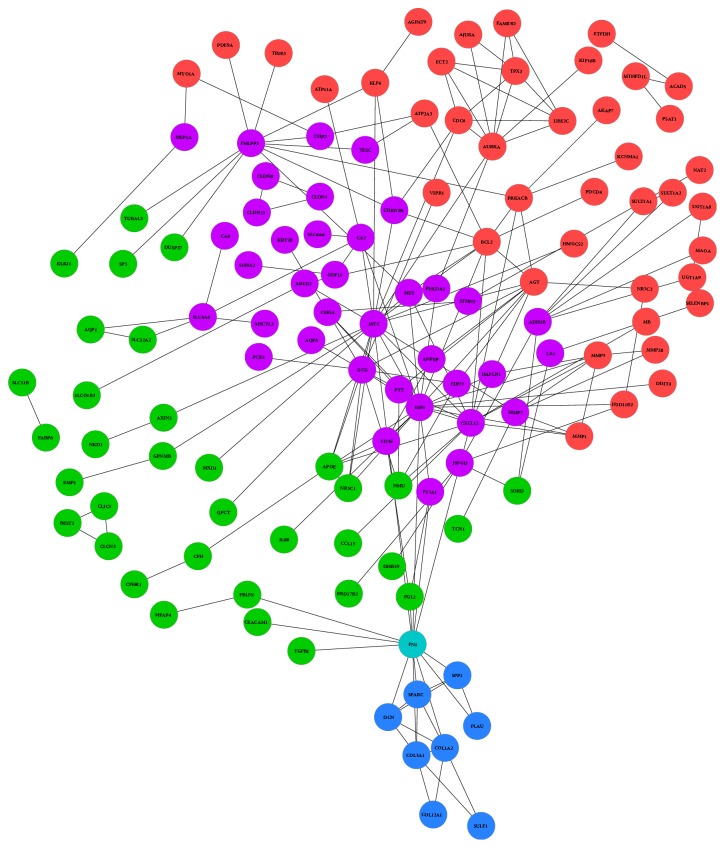
The protein-protein interactions (PPI) network of differentially expressed genes (DEGs) for each comparison. Red, green, and blue hubs represent carcinoma compared to normal, adenoma compared to normal, and carcinoma compared to adenoma, respectively. Purple hubs represent genes which are common to carcinoma compared to normal and adenoma compared to normal groups, while turquoise represents genes common to adenoma compared to normal and carcinoma compared to adenoma groups.

The PPI network was constructed on the basis of STRING database and visualized using Cytoscape software.  Figure 3 represents network of genes differentially expressed in our analysis. In the whole network, the top hub genes are IGF1 (21), MYC (20), FN1 (14), CXCL12 (14), GCG (13), AGT (10), and BCL2 (10). The number in brackets represents the number of interaction each gene has with other genes in network.

We identified top hub genes in each group, where there are at least four connections for a gene. In adenoma compared to normal top hub genes are APOE (7), NR3C1 (4), and NMU (4), in carcinoma compared to normal top hub genes are AGT (10), BCL2 (10), AURKA (9), MMP3 (6), CDC6 (6), TPX2 (6), PRKACB (6), UB2C (5), SULT1A1 (4), KLF4 (4), ECT2 (4), and MMP1 (4), and in carcinoma compared to adenoma top hub genes are COL3A1 (6), COL1A2 (6), SPARC (5), DCN (5), and SPP1 (4).

### 3.2. Functional Enrichment Analysis

The top five significant terms of GO and KEGG enrichment analysis are presented in Table 1, while all terms can be viewed in Supplementary Table 2. The group carcinoma compared to normal exhibits enrichment in biological process of regulation of protein phosphorylation, one-carbon metabolic process, anion transport, response to endogenous stimulus, and bicarbonate transport. As for molecular function, these genes are enriched in carbonate dehydratase activity, catalytic activity, hormone activity, binding, and metallopeptidase activity. Cellular function is enriched for genes which are included in extracellular region, vesicle, membrane-bounded vesicle, extracellular region part, and extracellular exosome. The biological processes enriched in adenoma compared to normal group were anion transport, one-carbon metabolic process, bicarbonate transport, organic anion transport, and ion transport. In this group, only one molecular function term was enriched, namely, carbonate dehydratase activity. Genes were enriched in cellular component of extracellular region, extracellular space, extracellular region part, membrane-bounded vesicle, and membrane region. It is interesting that in both described groups of carcinoma compared to normal and adenoma compared to normal the same KEGG pathways were enriched, i.e., nitrogen metabolism, bile secretion, and proximal tubule bicarbonate reclamation. Additionally, in cancer compared to normal group two more KEGG pathways were found, namely, chemical carcinogenesis and pancreatic secretion.

### 3.3. Carcinoma Compared to Adenoma

**Figure 4:**
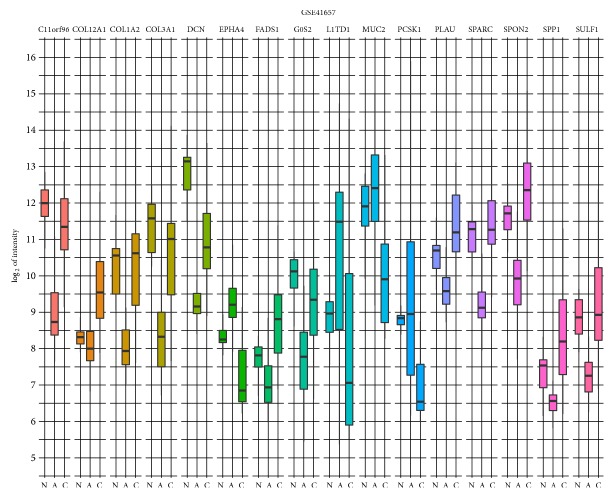
Logarithmic values of average intensities for normal (N), adenoma (A), and carcinoma (C) samples for GSE41657. Logarithmic values of average intensities for GSE37364, GSE10714, GSE50114, and GSE50115 can be found in Supplementary Figure 2.

The most interesting is the comparison between adenoma and carcinoma. Construction of contrast matrix enables us to compare the two groups, yet we have no information about the third group. To compare all three groups, we constructed a figure of logarithmic average intensity values, comparing normal, adenoma, and carcinoma samples (Figure 4). The figure shows that the 16 genes unique to carcinoma compared to adenoma group are also distinguishable from average intensities of normal samples. There are four types of changes in expression.* COL12A1* follows the first pattern and has similar expression in normal and adenoma, while in carcinoma the expression is elevated. The other pattern is that expression is similar in normal and adenoma, and reduced expression is observed in carcinoma. Genes that follow this pattern are* KIAA1324* and* PCKS1*.* EPHA4 *and* L1TD1* follow the third pattern, which higher expression in adenoma and lower in normal and carcinoma. All the other genes* C11orf96*,* COL1A2*,* COL3A1*,* DCN*,* FADS1*,* G0S2*,* PLAU*,* SPARC*,* SPON2*,* SPP1*, and* SULF1* follow the fourth pattern, where expression is decreased in adenoma and increased in normal and carcinoma.

## 4. Discussion

The CRC can arise through the progression of adenoma, which is the consequence of genetic and epigenetic events in epithelial cells. Some microarray studies have already identified gene expression profiles of adenoma and carcinoma [11, 19–24]. However, a study conducted by Nannini et al. revealed there is a rather weak overlap of gene expression profiles among different studies. They assigned this to several reasons: technical variability arising from collection of samples, protocols used for sample preparation, type of microarray used and subsequent data analysis pipeline used, and lack of large scale study [25]. We overcame some of these limitations by using more datasets on two different platforms, Affymetrix Human Genome U133 Plus 2.0 array and Agilent Whole Human Genome Microarray 4x44K G4112F. We used four raw datasets of microarray gene expression studies (GSE10714–Gambo et al. [19], GSE37364–Valcz et al. [26], GSE41657, GSE50114 and GSE50115–the latter three unpublished) and conducted our procedure of normalization, summation, and filtration, irrespective of procedures supplied by authors of the data.

The aim of this study was to investigate the differences in gene expression profiles of colorectal adenoma compared to adenocarcinoma, using normal mucosa samples as the reference. Our analysis showed many changes occur in adenoma compared to the normal group, suggesting that adenoma is an intermediate state between normal and carcinoma, although not all the changes found in carcinoma were found in adenoma. We identified 16 gene expression patterns unique to carcinoma compared to adenoma, suggesting that these 16 genes have a role in promoting progression of adenoma to carcinoma. Some of these genes have already been reported in adenoma compared to carcinoma, such as* SPON2* [15],* SPP1*, and* SPARC* [11], which is validation for our own analysis.

Functional analysis of genes in carcinoma compared to adenoma group revealed that the most significant biological processes and KEGG pathways are connected to extracellular matrix (ECM). Top two significant biological processes in this comparison are ECM disassembly and the other ECM organization; furthermore the top KEGG pathway is ECM-receptor interaction. Genes involved in these two biological process pathways are similar;* COL12A1*,* COL1A2*,* COL3A1*,* DCN*,* FN1*, and* SPP1* are involved in ECM disassembly and the same genes with addition of* SPARC* and* SULF1* are involved in ECM organization (Supplementary Table 2). Genes involved in these pathways are all upregulated in carcinoma compared to adenoma, indicating that the process of ECM organization is involved in the progression of adenoma to carcinoma.

The ECM is a superstructure, which has a supportive role, but on the other hand, it also delivers signals to cells, which determines their behavior. Therefore, the EMC is directly involved in process of EMT during malignant transformation and plays a major role in the pathology of cancer [27]. Results of our analysis show that nine out of 16 genes, which showed differential expression in carcinoma compared to adenoma, are components of ECM. These genes are all three collagen genes,* DCN, PLAU, SPARC, SPON2, SPP1*, and* SULF1*. They all showed an increase in expression in carcinoma compared to adenoma in our study.

Two collagen I proteins (*COL1A1*,* COL1A2*) were found significantly upregulated in cancer group compared to normal tissue. The study revealed higher expression of collagen I in stage II tumors, suggesting that the activation of collagen I is an early event in CRC progression. The finding suggests that expression of collagen I is higher at early stages of CRC and that collagen I is needed for tumor invasiveness [28]. Studies on cell lines suggest that adherence to collagen I promotes intracellular signaling pathways, including AKT pathways; furthermore collagen I was demonstrated to induce EMT-like changes, associated with tumor progression and metastasis [29, 30]. Expression of* COL3A1* gene was shown to be upregulated in CRC compared to normal controls. Wang et al. used Kaplan-Meier survival analysis to show that increased* COL3A1* protein in cancer epithelial cells predicted a worse prognosis [31]. The study was expanded to plasma samples, where soluble extracellular protein* COL3A1* was also significantly higher in patients with CRC compared to normal controls. Also,* COL3A1* was found to promote CRC cell proliferation by activating AKT signaling pathway [31]. One study used microarray data (GSE20219) and experimentally validated* COL12A1* gene. Its expression continuously increased from normal, through adenoma to carcinoma. Moreover, expression of* COL12A1 *was reported to clearly distinguish between normal, adenoma, and carcinoma group and may have further diagnostic potential [32]. Besides collagens, EMC contains also other proteins, such as proteoglycans, sulfatases, and phosphoproteins. DCN is a fibril-associated proteoglycan, found in EMC. Although upregulated when compared carcinoma to adenoma, the overall expression of* DCN* is downregulated when carcinoma to normal and adenoma to normal is compared. The role of* DCN* both in vivo and in vitro suggested that its role is tumor suppressive in stromal and epithelial cells [33]. A negative correlation between the immunoreactivity of* DCN* and malignant potential was observed [34].

The* PLAU* is a urokinase-like plasminogen activator (uPA), which is secreted serine protease that converts plasminogen into active plasmin. Binding of uPA to its receptor, uPA-R, activates its proteolytic activity, which promotes ECM degradation and subsequently the invasion and migration of tumor cells. The* PLAU* is found to be upregulated in CRC. Furthermore, increased activity of the plasmin/plasminogen system leads to tumor budding, which is also significantly related to lymph node metastasis [35]. SPARC is a member of the family of matricellular proteins, a calcium-binding protein. Studies have shown that* SPARC* expression in mesenchymal and stromal cells (MSC) was significantly higher compared to expression in cancer cells and in normal mucosa tissues. Low expression of* SPARC* is an independent unfavorable prognostic factor of colorectal cancer [36]. Another secreted ECM protein is SPON2, which belongs to mindin/F-spondin family. Spondin proteins play important role in different signaling pathways, important in cancer. SPON2 is found to be upregulated in many cancers, including CRC. SPON2 was also tested as a biomarker in plasma of CRC patients, where it was upregulated and downregulated after surgery was performed, indicating SPON2 to be associated with tumor burden [37]. SPP1 is phosphoprotein found upregulated in many cancers, including CRC. It was found to promote cell proliferation and metastasis by activating EMT [38]. The last ECM component that was significantly differentially expressed between adenoma and carcinoma is sulfatase,* SULF1. *Sulfatases are overexpressed in CRC and contribute to cell proliferation, migration, and invasion [39].

Other genes are not connected to ECM but are included in the progression of carcinoma. Two more genes had increased expression in carcinoma compared to adenoma, and these are* FADS1* and* G0S2. *FADS1 is a member of the fatty acid desaturase gene family and has been suggested to regulate inflammation by modifying the metabolite profiles of fatty acids, which may influence the progression of cancer. Decreased expression of FADS1 benefits development and growth of cancer cells, whereas increased expression was observed to be a protective factor in esophageal squamous cell carcinoma (ESCC). Decreased expression was associated with poor prognosis in patients with ESCC [40]. Expression of* G0S2* is downregulated in a wide variety of cancer cell types and has the properties of tumor suppressor. The upregulation of* G0S2* has shown a significant reduction in tumor cell growth and motility. Since the* G0S2* is a negative regulator of triglyceride catabolism, an altered lipid metabolism is present in transformation of cells from normal to cancerous [41].

The last four genes in our study were downregulated in carcinoma compared to adenoma, and those are* EPHA4, KIAA1324*,* L1TD1*, and* PCKS1*. Upregulation of EPHA4 was observed in various cancers, including CRC. The study shows that activated EPHA4 is associated with highly aggressive EMT-like phenotype. Also, activation of EPHA4 reduced E-cadherin expression and controlled cell migration and invasion through PI3K signaling [42]. The KIAA1324 is a transmembrane protein, also known as EIG121 (estrogen-induced gene 121). It was shown that KIAA1324 acts as a tumor suppressor in gastric cancer cell lines, where the induction of* KIAA1324* gene expression significantly reduced tumor size [43]. L1TD1 is RNA-binding protein, which is highly expressed in pluripotent cells. Depletion of L1TD1 leads to reduction in levels of OCT4 and NANOG and increased differentiation in human embryonic stem cells (hESCs). L1TD1 is required for self-renewal of hESCs and is reported as one of the key regulators of stem cell fate [44]. One study reported an increased expression of* PSKC1* in nasal polyps compared to the normal nasal mucosa. Furthermore, they showed that increased expression of* PSKC1* induces EMT-like process in airway epithelial cells. The cell lines displayed a morphological transformation from typical epithelial-like shape to an elongated, spindle morphology. The overexpression in* PSKC1* resulted in an enhanced cell proliferation and exhibits a significant increase in cell migration after wounding. Also the cells displayed reduced expression of epithelial markers and increased expression of mesenchymal markers [45].

**In conclusion, **distinguishing adenomas with epithelial misplacement (pseudoinvasion) from adenomas with early carcinomas (true invasion) is of great importance, in order to choose the optimal treatment. For this purpose, we identified 16 candidate genes with different expression patterns in adenoma compared to carcinoma, with a potential to discriminate between these two lesions, which will be the basis of our future work, where we will experimentally validate genes on selected tissue sections of adenomas with epithelial misplacement and adenomas with early carcinomas.

## Figures and Tables

**Table 1:** Gene ontology and Kyoto Encyclopedia of Genes and Genomes (KEGG) analysis of differentially expressed genes in each comparison group.

Pathway ID	Pathway description	Number of observed genes	FDR	Number of genes up/down regulated
**Carcinoma vs normal**				

**Biological process**				

GO.0001932	Regulation of protein phosphorylation	29	7.58E-05	16↑/13↓

GO.0006730	One-carbon metabolic process	7	7.58E-05	1↑/6↓

GO.0006820	Anion transport	17	0.000315	4↑/13↓

GO.0009719	Response to endogenous stimulus	30	0.000315	8↑/22↓

GO.0015701	Bicarbonate transport	6	0.000315	0↑/6↓

**Cellular component**				

GO.0005576	Extracellular region	63	9.38E-05	14↑/49↓

GO.0031982	Vesicle	53	0.000163	12↑/41↓

GO.0031988	Membrane-bounded vesicle	52	0.000163	12↑/40↓

GO.0044421	Extracellular region part	55	0.000163	13↑/42↓

GO.0070062	Extracellular exosome	42	0.00196	9↑/31↓

**Molecular function**				

GO.0004089	Carbonate dehydratase activity	5	0.0001	0↑/5↓

GO.0003824	Catalytic activity	70	0.000147	15↑/55↓

GO.0005179	Hormone activity	7	0.00231	2↑/5↓

GO.0005488	Binding	106	0.00306	35↑/71↓

GO.0008237	Metallopeptidase activity	9	0.00509	3↑/6↓

**KEGG**				

910	Nitrogen metabolism	5	5.28E-05	0↑/5↓

4964	Proximal tubule bicarbonate reclamation	4	0.00167	0↑/4↓

4976	Bile secretion	6	0.00167	0↑/6↓

5204	Chemical carcinogenesis	6	0.00167	0↑/6↓

4972	Pancreatic secretion	6	0.00513	0↑/6↓

**Adenoma vs normal**				

**Biological process**				

GO.0006820	Anion transport	15	5.23E-05	3↑/12↓

GO.0006730	One-carbon metabolic process	5	2.40E-03	0↑/5↓

GO.0015701	Bicarbonate transport	5	0.0024	0↑/5↓

GO.0015711	Organic anion transport	11	0.0024	3↑/8↓

GO.0006811	Ion transport	17	0.0359	3↑/14↓

**Cellular component**				

GO.0005576	Extracellular region	47	4.53E-06	10↑/37↓

GO.0005615	Extracellular space	23	1.15E-05	6↑/17↓

GO.0044421	Extracellular region part	41	1.15E-05	8↑/33↓

GO.0031988	Membrane-bounded vesicle	37	0.000128	6↑/31↓

GO.0098589	Membrane region	19	0.000128	5↑/14↓

**Molecular function**				

GO.0004089	Carbonate dehydratase activity	4	0.00115	0↑/4↓

**KEGG**				

910	Nitrogen metabolism	4	1.89E-04	0↑/4↓

4976	Bile secretion	6	0.000189	1↑/5↓

4964	Proximal tubule bicarbonate reclamation	4	0.000204	0↑/4↓

**Carcinoma vs adenoma**				

**Biological process**				

GO.0022617	Extracellular matrix disassembly	6	3.11E-05	6↑/0↓

GO.0030198	Extracellular matrix organization	8	3.11E-05	8↑/0↓

GO.0009888	Tissue development	11	0.0017	8↑/3↓

GO.0060279	Positive regulation of ovulation	2	0.0128	2↑/0↓

GO.0018149	Peptide cross-linking	3	0.0138	3↑/0↓

**Cellular component**				

GO.0005615	Extracellular space	14	4.03E-08	11↑/3↓

GO.0044420	Extracellular matrix component	6	5.01E-06	6↑/0↓

GO.0098644	Complex of collagen trimers	3	0.00151	3↑/0↓

GO.0005581	Collagen trimer	4	0.00166	4↑/0↓

GO.0044421	Extracellular region part	14	0.00637	10↑/4↓

**Molecular function**				

GO.0050840	Extracellular matrix binding	3	0.0478	3↑/0↓

**KEGG**				

4512	Extracellular-receptor interaction	4	0.00111	4↑/0↓

4510	Focal adhesion	4	0.0155	4↑/0↓

4974	Protein digestion and absorption	3	0.0155	3↑/0↓

5146	Amoebiasis	3	0.0219	3↑/0↓

4151	PI3K-Akt signaling pathway	4	0.0461	4↑/0↓

## Data Availability

The data used to support the findings of this study are available from the corresponding author upon request.

## Supplementary Materials

Supplementary 1 Supplementary Figures: Figure 1 shows the number of genes differentially expressed in each comparison and their intersection; Figure 2 shows the logarithmic values of average intensities for normal, adenoma, and carcinoma samples for (a) GSE10714, (b) GSE37364, (c) GSE50114 and GSE50115.

Supplementary 2 Supplementary Table 1 shows a list of genes common in each comparison and their expressions for each project.

Supplementary 3 Supplementary Table 2 shows gene ontology and KEGG of differentially expressed genes in each comparison group.
