# Supplementary material for: Bioinformatics Analysis Reveals Most Prominent Gene Candidates to Distinguish Colorectal Adenoma from Adenocarcinoma
Source: Biomed Res Int. 2018 Aug 6;2018:9416515. doi: 10.1155/2018/9416515 (PMC6106857; doi:10.1155/2018/9416515)
Supplement: Supplementary 1 — Supplementary Figures: Figure 1 shows the number of genes differentially expressed in each comparison and their intersection; Figure 2 shows the logarithmic values of average intensities for normal, adenoma, and carcinoma samples for (a) GSE10714, (b) GSE37364, (c) GSE50114 and GSE50115. [file 9416515.f1.docx]

**Supplementary Figures**


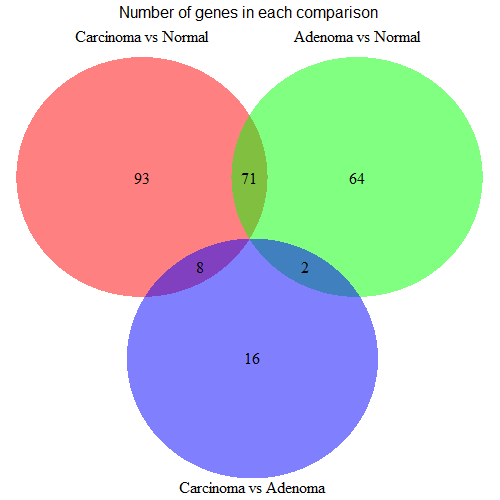


**Supplementary Fig. 1** Number of genes differentially expressed in each comparison and their intersection.


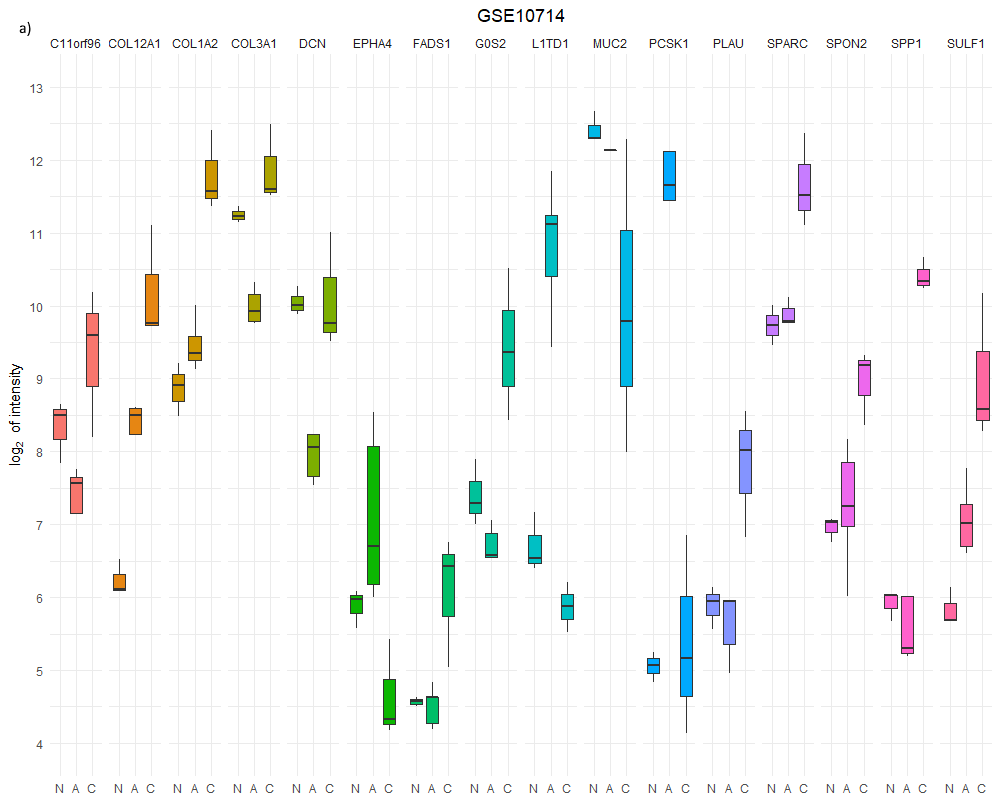


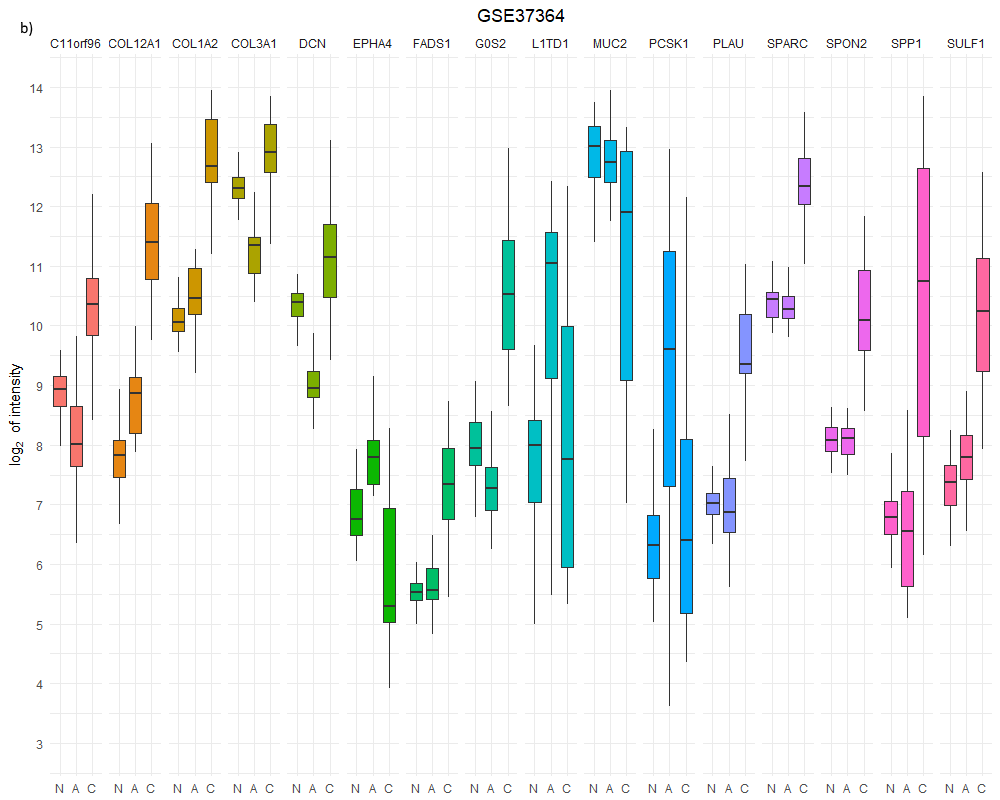


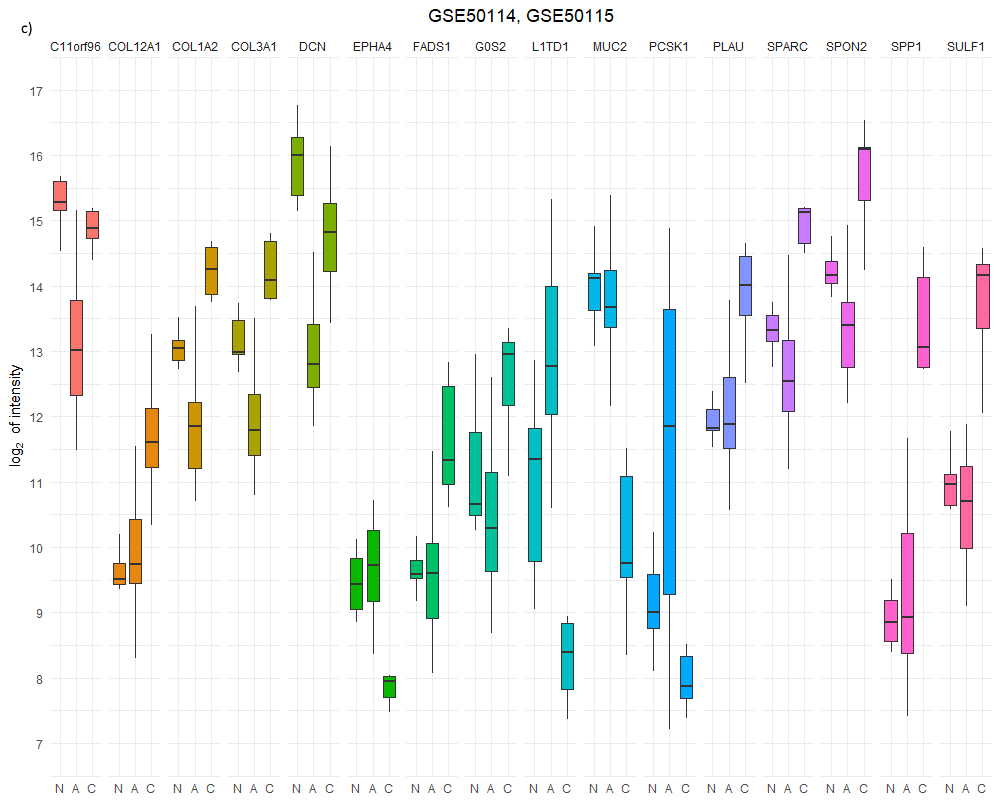


Supplementary Fig. 2 Logarithmic values of average intensities for normal (N), adenoma (A) and carcinoma (C) samples for a) GSE10714, b) GSE37364, c) GSE50114, GSE50115.
